# New Insights in (Poly)phenolic Compounds: From Dietary Sources to Health Evidence

**DOI:** 10.3390/foods9050543

**Published:** 2020-04-30

**Authors:** Raúl Domínguez-Perles, Nieves Baenas, Cristina García-Viguera

**Affiliations:** 1 Phytochemistry and Healthy Foods Lab, Department Food Science and Technology, CEBAS-CSIC, University Campus of Espinardo, 25, 30100 Espinardo, Murcia, Spain; rdperles@cebas.csic.es; 2 Department of Food Technology, Food Science and Nutrition, Faculty of Veterinary Sciences, University of Murcia, 30100 Espinardo, Murcia, Spain; nieves.baenas@um.es

**Keywords:** (poly)phenols, bioactivity, bioavailability, inflammation, secondary metabolites, antibacterial, antioxidant, diet, fruit, vegetables

## Abstract

Nowadays, there is a gap between the theoretical bioactivity of (poly)phenols and their real influence in health, once ingested. Due to this, new studies, including in vitro and in vivo models that allow for exploring bioaccessibility, bioavailability, and bioactivity, need to be developed to understand the actual importance of consuming functional foods, rich in these plant secondary metabolites. Moreover, current new strategies need to be developed to enhance the content of these foods, as well as setting up new formulations rich in bioaccessible and bioavailable compounds. Altogether, it could give a new horizon in therapy, expanding the use of these natural functional compounds, ingredients, and foods in the clinical frame, reducing the use of synthetic drugs. As a result, the joint contribution of multidisciplinary experts from the food science, health, and nutrition areas, together with the industrial sector, would help to reach these objectives. Taking this into account, diverse studies have been included in this study, which comprises different strategies to approach these objectives from different, complementary, points of view, ranging from the enrichment of by-products in bioactive compounds, through different agricultural techniques, to the assimilation of these compounds by the human body, both in vitro and in vivo, as well as by clinical studies.

To date, the biological interest of bioactive phytochemicals in plant-based foods is widely accepted. Indeed, (poly)phenolic compounds are of particular interest. These benefits have been associated with their antioxidant, antimicrobial, anti-inflammatory, anticancer, and cardiovascular protection activities, which support the application of these compounds in pharmaceutical, food, and cosmetic industries. Nonetheless, there are still some gaps related to the bioaccessibility, bioavailability, and bioactivity of these molecules, once ingested by the diet, that require additional studies contributing to clarify these issues, including in vitro and in vivo studies. Further efforts on clarifying these aspects closely linked to the biological benefits associated with (poly)phenols will allow us to develop an understanding of the actual scope of consuming functional foods that are rich in these plant secondary metabolites. In this frame, the present study gathers seven articles, six research papers and one review that provide further insight into the effect of manufacturing and biological processes on the actual biological interest of (poly)phenols.

The first approach to the main objective of this study comes from the new challenge that arises from the global climate changes, which modulates soil salinity and increases the demand for water. In this hallmark, Garcia-Campos et al. [1] focused the research on the management of plant physiology with beneficial bacteria, to obtain a biological tool that contributes to improving leaf bioactive profile and plant adaptation under saline stress in olive trees. In this chapter, the authors report the improvement of the (poly)phenolic composition of plant by-products at the same time as increasing the fitness of the tree when using a beneficial bacteria strain. In this respect, the described results evidence the capacity of such a bacterial strain to trigger plant metabolism that targets several mechanisms simultaneously, concluding that several strains increase osmoprotectant activity and photosynthesis in olive tree leaves, affect the enzymatic and non- enzymatic antioxidant system, and increases the content of bioactive (poly)phenols in leaves. Rendering, in the end, a promising by-product, with enriched health bioactive compounds.

With the same objective, González-Barrios et al. [2] focused their research on the production system, to augment the content of the bioactive (poly)phenols, in this case, on a manufactured food, chocolate, by the use of a cocoa powder rich in these bioactive compounds. As a result, the authors obtained a formulation for new dark chocolate, enriched in flavan-3-ols and oligomeric procyanidins, with acceptable organoleptic characteristics, and high antioxidant properties, describing the main improvements made to manufacturing in the chapter.

Another aspect covered in this study is the activity of certain foods on several diseases, in which evidence was retrieved resorting to in vitro systems studies. A study by Sánchez et al. [3] describes the use of a rich (poly)phenolic cranberry extract against periodontal pathogens, as an alternative to classical antibiotics that could contribute to prevent antibiotic resistance. To achieve this objective, the authors studied the anti-bacterial activity, applying a validated in vitro biofilm model. The obtained results indicate that cranberry (poly)phenolic compounds interfere in the phase of bacterial adherence, preventing colonization by inhibiting the adherence of these pathogens to oral tissues. Besides, these extracts could reduce the incidence and severity of periodontal disease-related symptoms, by suppressing inflammatory cascades, caused by the immunological response to bacterial infection.

Following the same guideline, Oliviero et al. [4] studied the influence of polydatin and resveratrol, in the reduction of the inflammatory process induced by monosodium urate and calcium pyrophosphate in type-1 cells, demonstrating that both phenolic compounds are effective in inhibiting reactive oxygen species (ROS) and nitric oxide (NO) production, as well as crystal-induced inflammation. Due to this, the authors conclude that dietary supplementation with these compounds could support pharmacological treatments in patients affected by this inflammatory process and thus prevent the acute phase of the disease.

Likewise, studies of the efficacy or bioavailability of these compounds are also included in the present study, by the hand of diverse human clinical studies, concerning obesity. These studies are collected in two additional chapters. On one hand, Marhuenda et al. [5], have paid attention to the potential use of (poly)phenols to reduce obesity, studying the effectiveness of a blend formulated with *Hibiscus sabdariffa* and *Lippia citriodora* extracts for the treatment of obesity and/or overweight control in the absence of a controlled diet. The results demonstrate the association between the consumption of this blend and the reduction in body weight, Body Mass Index (BMI), and central fat mass, due to the possible changes in molecular pathways. On the other hand, Agulló et al. [6] have characterized a total of 16 flavanone urine metabolites, after the ingestion of a new maqui/citrus beverage, sweetened with sucrose (natural and high caloric), stevia (natural and low caloric) or sucralose (artificial and low caloric). The purpose of this study was to determine the influence of sweeteners in obesity, when added to a functional beverage, evaluating their possible interference with flavanones (phenolic bioactive compounds). The results indicate that both non-caloric additives provided a significantly higher urinary excretion for most compounds when compared to the traditional sweetener, sucrose. On this base, sucrose consumption could be reduced due to its evident influence on metabolic disorders, without providing any extra benefit in the absorption of bioactive (poly)phenols.

Finally, Carrillo et al. [7], reviewed the relationship between cognitive function and consumption of fruit and vegetable (poly)phenols in a young population, concluding that there is a positive effect of (poly)phenols related to neuroprotection and associated with the antioxidant and anti-inflammatory capacity of these compounds. This evidence emphasizes the importance of the intake of fruits and vegetables in maintaining normal cognitive function. Due to this fact, these bioactive compounds could also be considered nutraceutical supplements, useful as an alternative preventive therapy due to the lack of effective pharmaceutical treatments for age-related cognitive decline, implementing primary prevention actions in young people to minimize the cognitive impairment caused by age.
